# Amino Acid Polymorphisms in the VHIID Conserved Motif of Nodulation Signaling Pathways 2 Distinctly Modulate Symbiotic Signaling and Nodule Morphogenesis in *Medicago truncatula*

**DOI:** 10.3389/fpls.2021.709857

**Published:** 2021-12-13

**Authors:** Szilárd Kovacs, Lili Fodor, Agota Domonkos, Ferhan Ayaydin, Krisztián Laczi, Gábor Rákhely, Péter Kalo

**Affiliations:** ^1^Institute of Plant Biology, Biological Research Center, Eötvös Lóránd Research Network, Szeged, Hungary; ^2^Institute of Genetics and Biotechnology, Hungarian University of Agriculture and Life Sciences, Gödöllö, Hungary; ^3^Hungarian Centre of Excellence for Molecular Medicine (HCEMM) Nonprofit Ltd., Szeged, Hungary; ^4^Cellular Imaging Laboratory, Biological Research Center, Eötvös Lóránd Research Network, Szeged, Hungary; ^5^Department of Biotechnology, University of Szeged, Szeged, Hungary; ^6^Institute of Biophysics, Biological Research Center, Eötvös Lóránd Research Network, Szeged, Hungary

**Keywords:** root nodule symbiosis, nitrogen fixation, nod-factor signaling, *Medicago*, rhizobia, GRAS transcription factors, NSP1, NSP2

## Abstract

Legumes establish an endosymbiotic association with nitrogen-fixing soil bacteria. Following the mutual recognition of the symbiotic partner, the infection process is controlled by the induction of the signaling pathway and subsequent activation of symbiosis-related host genes. One of the protein complexes regulating nitrogen-fixing root nodule symbiosis is formed by GRAS domain regulatory proteins Nodulation Signaling Pathways 1 and 2 (NSP1 and NSP2) that control the expression of several early nodulation genes. Here, we report on a novel point mutant allele (*nsp2-6*) affecting the function of the *NSP2* gene and compared the mutant with the formerly identified *nsp2-3* mutant. Both mutants carry a single amino acid substitution in the VHIID motif of the NSP2 protein. We found that the two mutant alleles show dissimilar root hair response to bacterial infection. Although the *nsp2-3* mutant developed aberrant infection threads, rhizobia were able to colonize nodule cells in this mutant. The encoded NSP2 proteins of the *nsp2-3* and the novel *nsp2* mutants interact with NSP1 diversely and, as a consequence, the activation of early nodulin genes and nodule organogenesis are arrested in the new *nsp2* allele. The novel mutant with amino acid substitution D244H in NSP2 shows similar defects in symbiotic responses as a formerly identified *nsp2-2* mutant carrying a deletion in the *NSP2* gene. Additionally, we found that rhizobial strains induce delayed nodule formation on the roots of the *ns2-3* weak allele. Our study highlights the importance of a conserved Asp residue in the VHIID motif of NSP2 that is required for the formation of a functional NSP1-NSP2 signaling module. Furthermore, our results imply the involvement of NSP2 during differentiation of symbiotic nodule cells.

## Introduction

The restricted availability of nitrogen extremely restrains plant development. *Medicago truncatula* and other leguminous plants can overcome this limitation because of their capability to establish nitrogen-fixing symbiotic associations with soil bacteria collectively termed as rhizobia. Symbiotic nodules are specialized organs that formed on legume roots, which provide a microaerobic environment for endosymbiotic rhizobia. Within the nodules, rhizobia reduce atmospheric nitrogen to ammonia that is utilized by the host plant in return to carbon sources (Gibson et al., 2008). Nodule formation is a result of several consecutive communication steps between the two organisms. The bacterially derived lipochitooligosaccharides called Nod factors (NFs) are key signaling molecules for the early stages of nitrogen-fixing symbiotic interactions [reviewed in references (Jones et al., 2007; Oldroyd and Downie, 2008)].

The perception of NFs induces a signaling pathway that activates many early symbiotic responses in the symbiosis sensitive region of the host root. NFs trigger root hair deformation, oscillations in cytosolic calcium levels (Ca-spiking) in root hair cells, and induction of symbiosis specific gene expression (epidermal responses). NFs also induce mitotic activation of inner cortical cells that leads to the formation of nodule primordia (cortical response) (Oldroyd and Downie, 2008). Genetic mutants of the model legumes *M. truncatula* and *Lotus japonicus* defective in the transduction of the rhizobial symbiotic signal were used to identify the components of the NF signaling pathway, such as receptors, signal-transmitting kinases such as DMI2 (Endre et al., 2002) [SymRK in *L. japonicus* (Stracke et al., 2002)], ion-channel proteins, and transcription factors (Oldroyd, 2013; Kang et al., 2016). A calcium/calmodulin-dependent protein kinase (CCaMK) interacting with the IPD3 (CYCLOPS in *L. japonicus*) transcriptional activator (Singh et al., 2014) decodes the calcium signal and regulates both epidermal and cortical responses (Oldroyd and Downie, 2008), which are mediated by GRAS proteins, Nodulation Signaling Pathways 1 and 2 (NSP1 and NSP2) (Kalo et al., 2005; Smit et al., 2005; Heckmann et al., 2006) and other transcriptional regulators [e.g., NIN, ERNs, and NF-YA (Schauser et al., 1999; Andriankaja et al., 2007; Middleton et al., 2007; Laporte et al., 2014)]. The NSP1–NSP2 complex interacts with IPD3 and CCaMK through DELLA proteins (Jin et al., 2016).

Nodulation Signaling Pathways 1 and 2 are GRAS-type transcription regulators named after the first three discovered members, *GIBBERELLIN-INSENSITIVE* (*GAI*), repressor of *ga1-3* (*RGA*), and *SCARECROW* (*SCR*) (Pysh et al., 1999), and are essential for most rhizobium-induced symbiotic responses. Mutant plants defective in NSP1 or NSP2 show altered root-hair deformation and are blocked in NF-induced gene expression, infection thread (IT) initiation, and cortical cell division (Catoira et al., 2000; Oldroyd and Long, 2003). The NSP2 protein plays a critical role in the activation of early nodulin gene expressions, such as *MtENOD11* expression, in the epidermis through NF signaling (Gleason et al., 2006). NSP2 is also essential for the CCaMK-activated cytokinin signaling pathway *via* the cytokinin receptor CRE1 leading to cortical cell division and nodule organogenesis (Tirichine et al., 2007; Ariel et al., 2012). NSP2 forms a heterodimer with NSP1, and the complex of NSP2 and NSP1 directly binds to promoters of early nodulin genes, such as *ENOD11*, *ERN1*, and *NIN* (Hirsch et al., 2009). The GRAS domains of NSP1 and NSP2 contain two leucine heptad repeat motifs (LHRI and LHRII) and have three additional motifs, VHIID, PFYRE, and SAW named after the most prominent residues, which are all of them characteristic for GRAS proteins (Bolle, 2004). An analysis of deletion mutants of *NSP1* and *NSP2* revealed that several domains of NSP1 are required for the interaction with NSP2, and also showed that the LHR1 and VHIID of NSP2 domains have a prominent function in the association with NSP1 (Hirsch et al., 2009). The *nsp2-3* point mutant allele carrying the nonconservative substitution E232K develops small white nodules, indicating that the nodule developmental program is not completely blocked in this mutant (Kalo et al., 2005). The mutation of E232K abolishes the autoactivation of NSP2 but does not negatively affect the formation of the NSP1-NSP2 complex (Hirsch et al., 2009).

Bacterial infection is associated with root hair curling and formation of tubular structures called ITs in root hairs that guide rhizobia into the root cortex where bacteria invade cells of nodule primordia through an endocytosis-like process (Gage, 2002, 2004). Rhizobia are surrounded by plant–derived membranes forming a cytoplasmic structure referred to as symbiosomes (Brewin, 2004; Jones et al., 2007). Bacteria undergo morphological changes and metabolomic transition within symbiosomes (Pfau et al., 2018). Differentiated rhizobia termed bacteroids convert the atmospheric nitrogen in nodule cells into ammonia that can be assimilated by the host plant. The symbiotic interaction between *Sinorhizobium* (*Ensifer*) sp. and *M. truncatula* leads to the formation of cylindrical-shaped indeterminate nodules. The persistent meristem (zone I) of indeterminate nodules produces a developmental gradient of cells from the apex of nodules toward the senescent cells proximal to the root (Vasse et al., 1990). The subsequent zone to the meristematic region is the infection zone (zone II) wherein bacteria are released from ITs and internalized in symbiosomes that multiply, thereby filling up the colonized cells. Both rhizobia and infected nodule cells undergo differentiation in the interzone (zones II–III), resulting in enlarged infected cells containing elongated polyploid bacteroids. The nitrogen fixation zone (zone III) is composed of the central tissue of the indeterminate nodules. Zone III consists of small noninfected cells and large infected cells with differentiated bacteroids, which convert atmospheric nitrogen to ammonia. In the basal part of older nodules, bacteroids and plant cells are disintegrated and collapse in the senescence zone (zone IV) proximal to zone III.

The activity of the NF signaling pathway is not restricted to the initiation of infection and nodule organogenesis but also plays a role in later stages of nodule development. It was found that at least two NF signaling genes, *DMI2*, coding for a leucine-rich-repeat-containing receptor kinase (Endre et al., 2002), and *NIN*, coding for a transcription factor (Schauser et al., 1999), are expressed in the distal part of symbiotic nodules (Limpens et al., 2005; Liu et al., 2021). *DMI2* transcripts are abundant in few cell layers adjacent to the meristem, and *NIN* is highly expressed in the infection zone. It was demonstrated that the partial reduction of *DMI2* expression in wild-type plants or the misregulation of *DMI2* in *dmi2* background caused extensive IT formation and defects in symbiosome formation (Limpens et al., 2005). In two *nin* alleles of *M. truncatula*, *NIN* mRNA levels were greatly reduced but not abolished completely. In these *nin* mutants, nodule development was not blocked fully, which is in contrast with the non-nodulation phenotype of *nin* alleles wherein *NIN* transcripts were completely abolished. The mutant alleles that expressed the *NIN* gene at a low level developed white nonfunctional nodules showing early senescence, incomplete rhizobial differentiation, and defects in symbiosome formation (Liu et al., 2021). These findings indicated that some alleles of genes involved in the NF signaling pathway could reveal the function of these components in later stages of nodule development.

Several mutant alleles of *NSP2* were found previously in genetic screens of *M. truncatula* targeting the identification of symbiotic nitrogen-fixing mutants impaired in bacterial signal perception and transduction. The *nsp2* mutants were identified in mutant collections generated by either fast neutron (FN) bombardment [*nsp2-1*, *nsp2-2*, and *nsp2-5* alleles (Oldroyd and Long, 2003; Domonkos et al., 2013) or retrotransposon insertion (*nsp2-4* and several other insertions alleles (Rakocevic et al., 2009; Pislariu et al., 2012)] that generally generate knockout (null) mutants. The formerly identified *nsp2-3* mutant is able to form functional nodules with a symbiotically effective strain of rhizobia, and these nodules display unusual nodule colonization with a reduced number of uninfected cells.

In this study, we have analyzed two-point mutant *nsp2* alleles that carry substitutions in the VHIID motif of NSP2. The early steps of nodule development are completely blocked, and the interaction with NSP1, which is required for NF inducible gene activation, is abolished in the novel *nsp2-6* allele, supporting that the substitution in a conserved residue of the VHIID domain is essential for NSP2 function. We also report on a strain-dependent nodulation phenotype of the weak *nsp2-3* allele.

## Materials and Methods

### Plant Material, Bacterial Strains, and Growth Conditions

The *M. truncatula* Jemalong A17 genotype was used as a wild-type (wt) control in all of the experiments. The NF-FN9199 mutant line was identified in a former genetic screen (Kontra-Kováts et al., 2019). The *Mtnsp2-2* and *nsp2-3* mutant lines used in this study have been described previously (Kalo et al., 2005). Seeds were germinated as described in the *M. truncatula* handbook (Garcia et al., 2006). Seedlings were grown under symbiotic conditions in a zeolite substrate (Geoproduct Kft., Mád, Hungary) in growth chambers and kept under a photoperiod of 16 h of light and 8 h of darkness at 24°C. Five-day old seedlings were inoculated with a suspension of rhizobia diluted to OD_600_ 0.1 *Sinorhizobium* (*Ensifer*) *medicae* WSM419 carrying the pMEpTrpGFPGUS plasmid expressing the β-glucuronidase-GFP fusion protein (Auriac and Timmers, 2007), and strains *Sinorhizobium* (*Ensifer*) *meliloti* 1021 and *S. medicae* WSM419 carrying the pXLGD4 plasmid expressing the *lacZ* reporter gene under the control of the *hemA* promoter were used for inoculation of plants.

In hairy root transformation and *Nicotiana benthamiana* infiltration experiments, *Agrobacterium rhizogenes* ARqua1, and *Agrobacterium tumefaciens* C58C1 strains carrying appropriate constructs were used. All the bacterial strains were grown for 48–72 h at 30°C in a complete TA medium supplemented with appropriate antibiotics.

### Generation of Constructs

The constructs used in this study were generated with Gateway Technology (Invitrogen, Life Technologies, Carlsbad, CA, United States) according to the protocol of the manufacturer.

The mutant *NSP2* alleles were amplified from the genomic DNA of *nsp2-3* and *nsp2-6* using Q5High Fidelity DNA Polymerase (NEB, Ipswich, MA, United States) with the primers listed in Supplementary Material. An additional A nucleotide was added to the 3′ end by incubation with DreamTaq polymerase (Thermo Fisher Scientific, Waltham, MA, United States). A-tailed PCR fragments were then cloned into a pCR^TM^8/GW/TOPO^®^ (Invitrogen, Carlsbad, CA, United States) vector and verified by sequencing. Destination clones were generated by LR clonase II-(Invitrogen) mediated recombination using the entry constructs. For yeast two-hybrid (Y2H) experiments, destination vectors pDEST-GBKT7 and pDEST-GADT7 (Clontech Laboratories, Inc., Takara Bio Europe, Saint-Germain-en-Laye, France), and pUBN-cYFP-DEST (Grefen et al., 2010) plasmids were used for bimolecular fluorescence complementation (BiFC) assays. For genetic complementation experiments, a previously described construct consisting of the *NSP2* coding sequence under the control of the 35S constitutive promoter was used (Kalo et al., 2005).

The *NSP1* and *NSP2* expression clones (either for BiFC or Y2H) and the autoactive CRE1 clone were kindly provided by Giles Oldroyd (Cambridge University, United Kingdom) and Erik Limpens (Wageningen University, Netherlands).

### Yeast Two-Hybrid Experiments

Bait and prey plasmids were transformed into *Saccharomyces cerevisiae* PJ-69-4A cells as recommended by the manufacturer (Clontech Laboratories, Inc., Protocol No. *PT3247-1*. 2.) using the original components and salmon sperm DNA (Sigma) as carrier. Transformed PJ-69-4A yeast strains were selected on plates containing synthetic-defined (SD) minimal yeast media [SD (dropout)/without Leu and Trp (-LW)]. Expression of the *HIS3* reporter gene were tested using a spot assay. PJ-69-4A yeasts containing the appropriate constructs were grown in SD-LW medium with shaking at 30°C for 16 h. The diluted cultures (OD_600_ 0.1) were spotted to SD-LW with or without histidine (SD-HLW) containing 10 or 40 mM 3-amino-1,2,4-triazole (3-AT) plates for monitoring of the growth of yeast colonies. The liquid β-galactosidase assay was performed according to the Yeast Protocols Handbook (PT3024-1, Clontech). The immunoblot experiment was carried out to detect the stability of wild-type and mutant NSP2 proteins fused to the HA-tagged activation domain in yeast using an *a*-HA antibody according to the protocol of the manufacturer (Invitrogen, cat. 26183).

### Complementation and Bimolecular Fluorescence Complementation Experiments

The constructs generated for plant transformation experiments were introduced into *A. tumefaciens* C58C1 or *A. rhizogenes* ARqua1 strains by electroporation.

The hairy root transformation was carried out as described in The *Medicago truncatula* Handbook (Chabaud et al., 2006), and transgenic roots were selected based on the fluorescent signals of the DsRED marker protein.

For the infiltration of *N. benthamiana* leaves, two to three colonies of *A. tumefaciens* carrying paired vector combinations for the BiFC experiments and of the strain carrying the p19 silencing suppressor construct (Silhavy et al., 2002; Voinnet et al., 2003) were inoculated into 10 ml of TA medium and grown at 28°C with shaking for 16 h, and 1 ml of overnight bacterial culture was transferred into 20 ml fresh TA medium containing 10 mM MES (pH 5.6), 20 μM acetosyringone (Merck KGaA, Darmstadt, Germany), and appropriate antibiotics. After growth for 16 h at 28°C, the bacterial cultures were centrifuged at 4,000 rpm for 15 min. The pellet was resuspended in sterile water containing 10 mM MES (pH 5.6), 10 mM MgCl_2_, and 150 μM acetosyringone (MMA solution) diluted to OD_600_ 0.3 and incubated for 3 h at room temperature in the dark. The prepared *Agrobacterium* dilutions were mixed and infiltrated into the top leaves of 6-week-old *N. benthamiana* plants. Infiltration of the fully expanded leaves was performed with a 1-ml syringe, and the plants were kept in the dark overnight to enhance the efficiency of transformation. YFP fluorescence was observed 3 days after the infiltration by confocal laser scanning microscopy (see below).

### Histochemical Staining and Imaging

The nodule and root samples were harvested at indicated time points upon inoculation with rhizobia. For GUS staining, the nodules were embedded in 5% agarose, and 70 μm sections were prepared using a MICROM HM 650 V vibratome. Nodule sections or whole roots were vacuum-infiltrated for 30 min, and then incubated in a GUS staining solution [2 mM 5-bromo-4-chloro-3-indolyl β-D-glucuronide (X-Gluc; Duchefa Biochemie, Haarlem, The Netherlands), 1 mM EDTA, 0.1% Triton X-100, 0.1% Sarcosyl, 0.05% SDS, 1 mM potassium ferricyanide, and 1 mM ferrocyanide in phosphate buffered saline (PBS) (pH 7.4)] for 1–3 h at 37°C.

For β-galactosidase and SYTO13 (Invitrogen) staining, the nodule samples were vacuum-fixed in 4% paraformaldehyde three times for 30 s, post-fixed for 30 min on ice, and then washed 3 × 5 min in PBS (pH 7.4). The fixed samples were embedded in 5% (w/v) agarose, and 70 μm sections were prepared using a MICROM HM 650 V vibratome. To assay β-galactosidase activity, the sections were stained with 0.1% X-Gal in PBS (pH 7.4) supplemented with 5 mM potassium ferricyanide and 5 mM potassium ferrocyanide for 1–3 h at 37°C. The GUS and X-gal-stained samples were analyzed using DM LB2 Light Microscope (Leica), and images were captured using a QImaging MicroPublisher 3.3 RTV camera. For SYTO13-staining, the nodule sections were incubated in 1 × PBS (pH 7.4) containing 5 μM SYTO13 for 20 min at room temperature in the dark and then rinsed with 1 × PBS. Images were captured by confocal laser scanning microscopy.

### Confocal Laser Scanning Microscopy and Image Analysis

The samples were analyzed using an Olympus Fluoview FV1000 confocal laser scanning microscope (Olympus Life Science Europa GmbH, Hamburg, Germany). The microscope configuration was as follows: Objectives: UPLSAPO 10× dry (N.A. 0.4), UPFLN 40× Oil (N.A. 1.3), LUMPFLF 40× Water (N.A. 0.8); sampling speed: 8 μs/pixel; line averaging: 2; laser excitation: 405 (cell wall autofluorescence, pseudocolored red), 488 (SYTO 13, green), and 515 nm (YFP/BiFC, green). Autofluorescence was detected between 425 and 475 nm, and SYTO13 and YFP emissions were detected between 500 and 600, and 530 and 630 nm, respectively, using spectral detectors. Brightfield images were captured on a transmitted light detector by either 405- or 515-nm laser excitation.

To quantify the degree of nodule colonization, the FIJI software (ver. 1.53c) was used (Schindelin et al., 2012). Green colored SYTO13 images were converted to 8-bit black and white images. The perimeter of the nodules was traced manually using a freehand selection tool. Regions of interest were cut out and pasted as a new image to obtain a clean image while excluding any debris or non-cellular area around the nodules. To eliminate bias, the images were auto-thresholded to isolate and highlight highly fluorescent, infected cell-containing regions. Using the “Find connected regions” plugin of the FIJI program, the size and number of connected regions larger than 70 pixels were quantified. (Plugin was written by Mark Longair; for details of the FIJI plugin, visit: https://github.com/fiji/VIB/blob/master/src/main/java/util/Find_Connected_Regions.java).

A lower limit of 70 pixels was selected to include the smallest infected cells while excluding small fluorescent objects and image noise. Results displaying “the total number and size of the connected areas” were exported to Microsoft Excel to plot the charts after sorting areas from lowest to highest. Large connected areas (which were less in number) were considered as an indicator of more densely infected nodules, whereas smaller sized connected areas, which were also more frequent, were considered as an indicator of less frequently infected cells within the nodule.

### Acetylene Reduction Assay

The activity of bacterial nitrogenase was measured by acetylene reduction assay (ARA). Nodulated root sections of the mutant and wild-type plants were harvested 21 days after inoculation with either strain *S. meliloti* 1021 or strain *S. medicae* WSM419. Five to six nodules and root samples as negative control were enclosed in 1.8-ml glass vials sealed with rubber stoppers. Promptly, 180 ml acetylene (Linde Gas Hungary Co., Ltd., Répcelak, Hungary) was injected into the vials, the samples were incubated for 2 h in the dark, and then 100 ml gas was injected into an Agilent 6890 (Agilent Technologies Inc., Santa Clara, CA, United States) gas chromatograph equipped with a split/splitless injector and a Flame Ionization Detector to measure the amount of ethylene. The analytical column was HP-PLOT/Q+PT Cat No. 19095P-QO4PT (Agilent Technologies Inc.) with dimensions of 30 m × 0.53 mm × 40 μm. The carrier gas was hydrogen with a purity of 5.0 (Messer Hungarogáz Ltd., Budapest, Hungary). The column flow was 4.5 ml/min, and the split ratio was set to 9:1. The oven temperature was constant 50°C. The samples were injected manually with a 250-μl Hamilton syringe 1725 RN (Hamilton Company, Bonaduz, Switzerland) equipped with a ga22s pt5 needle. The effectiveness of acetylene reduction was calculated in ARA units (nmol of ethylene per mg nodule per hour). The ARA was carried out with four biological replicates. For ethylene calibration, ethylene gas (Linde Gas Hungary Co., Ltd.) with a purity of 3 was used. The molarity of ethylene was calculated with the van der Waals equation.

### Gene Expression Analysis

For real-time RT-PCR, root segments susceptible to rhizobia were harvested in liquid nitrogen at 4 days post inoculation (dpi) with *S. medicae* strain WSM19. RNA was extracted with TRI Reagent (Merck KGaA, Darmstadt, Germany) and Direct-zol RNA MiniPrep Kit (Zymo Research, Irvine, CA, United States). RNA samples were treated with DNaseI on columns according to the instructions of the manufacturer to remove the residual genomic DNA. Total RNA was quantified using a spectrophotometer (Nanodrop-1000; NanoDrop Technologies, Thermo Fisher Scienific, Waltham, MA, United States), and the quality was checked by gel electrophoresis. Complementary DNA was prepared from 1 μg total RNA with SuperScript IV First-Strand Synthesis System (Life Technologies, Invitrogen, Carlsbad, CA, United States) using oligo-dT primers according to the instructions of the manufacturer. Quantitative PCR (qPCR) was performed with LightCycler 96 (Roche, Mannheim, Germany) using qPCRBIOSyGreen Mix (PCR Biosystems, London, United Kingdom) according to the protocol of the manufacturer. Cycle threshold values were obtained and data were analyzed with the LightCycler 96 SW1.1 software. Relative expression levels were calculated by normalization against the expression of an ubiquitin-like gene (see Supplementary Table 1; Kakar et al., 2008). Values of relative transcript levels were the mean of five or six biological replicates. Fold induction was calculated by normalizing the data to the wild-type samples (A17 4 dpi). The primers used for qPCR are listed in Supplementary Table 1.

## Results

### The Conserved VHIID Motif Is Affected in Point Mutant Alleles of *NSP2*

**FIGURE 1:**
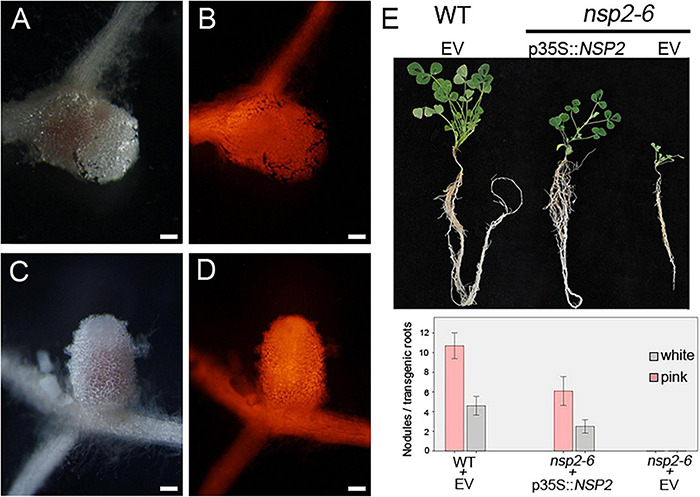
*NSP2* restores the nodulation phenotype of the *nsp2-6* allele. The *NSP2* gene driven by the constitutive 35S promoter (*p35S::NSP2*) was introduced into the *nsp2-6* roots by *Agrobacterium rhizogenes*-mediated hairy root transformation. Transformed roots were inoculated with *Sinorhizobium medicae* WSM419. Bright field stereomicroscope images of nodules **(A,C)** developed on transgenic roots identified by DsRed fluorescence **(B,D)** were analyzed at 21 days post inoculation with rhizobia. Nodules formed on the *nsp2-6* roots transformed with the *p35S::NSP2* construct **(A,B)**. Pink and nitrogen-fixing nodules were formed on wild-type (WT) *Medicago truncatula* A17 roots transformed with empty vector (EV) **(C,D)**. Retarded shoot growth of *nsp2-6* is partially restored with *p35S::NSP2* **(E)**. Average number of pink and white nodules detected on transgenic roots (WT *n* = 14, *nsp2-6 n* = 18 and 22 transformed with *p35S::NSP2* and empty vector, respectively **(E)**. Error bars represent standard errors. Scale bars, 200 μm **(A–D)**.

**FIGURE 2:**
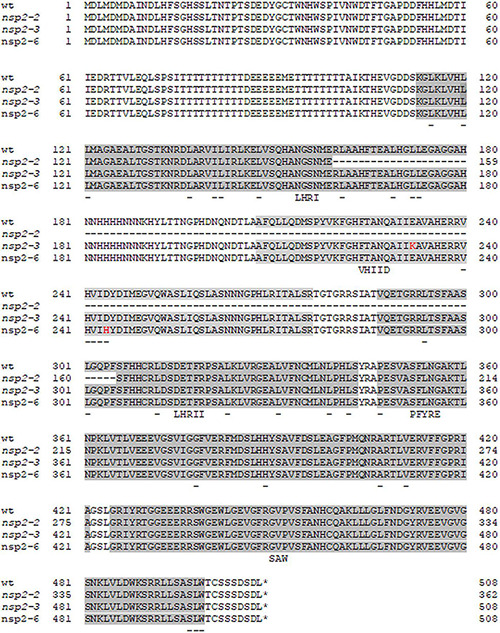
Alignment of the amino acid sequences of wild-type and Nodulation Signaling Pathway 2 (NSP2) mutant alleles. Single amino acid substitutions in the *nsp2-3* (E232K) and *nsp2-6* (NF-FN9199) (D244H) mutants are highlighted with red characters. The conserved LHRI, VHIID, LHRII, PFYRE, and SAW motifs of the GRAS domain are highlighted with gray background. Conserved residues of the subdomains are underlined. Deletion in *nsp2-2* allele affects the motifs LHRI, VHIID, and LHRII.

Nodulation Signaling Pathway 2 is an essential component of the NF signaling pathway (Oldroyd and Long, 2003). In order to investigate the altered activity and function of the NSP2 protein, formerly identified point mutant alleles of *NSP2* were analyzed in this study. The previously described point mutant allele *nsp2-3* containing a non-conservative E232K change forms small white nodules following inoculation with *S. meliloti* strain 1021 (Kalo et al., 2005), indicating impaired symbiosis. Another mutant, NF-FN9199, was identified in a forward genetic screen and was found to be completely deficient in nodule formation (Nod-) (Kontra-Kováts et al., 2019). The position of the nodulation defective locus and the sequence analysis of *NSP*2 in mutant NF-FN9199 suggested that this mutant is a novel *nsp2* allele. In order to confirm that the mutation in *NSP2* caused the nodulation deficient phenotype of NF-FN9199, genetic complementation experiments were carried out. The coding sequence of *NSP2* under the control of the 35S promoter was introduced into the NF-FN9199 roots by *A. rhizogenes*-mediated hairy root transformation. Transformed roots were detected by the DsRed fluorescent marker. The growth habit and formation of nodules on the transformed roots were assessed 3 weeks post inoculation (wpi) with *S. medicae* strain WSM419 (pXLGD4) (SmWSM419). NF-FN9199 mutant plants transformed with the *NSP2* gene showed improved growth rate and developed dark green leaves compared with empty vector-transformed mutant plants, indicating the recovery of mutant plants from nitrogen starvation (Figure 1E). Moreover, pinkish nodules that formed on the NF-FN9199 roots and transformed with *p35S::NSP2* also confirmed the function of symbiotic interaction with rhizobia (Figures 1A,B,E). Although nodules on the NF-FN9199 roots were less elongated compared with the nodules that formed on wild-type roots transformed with empty vector (Figures 1C,D), they were pink because of the presence of leghemoglobin, indicating the establishment of an efficient symbiotic interaction to some degree. However, the lower ratio of the pink nodules that formed on the NF-FN9199 roots transformed with the construct *p35S::NSP2* was observed compared with the empty vector-transformed wild-type roots (Figure 1E), indicating that the *NSP2* gene controlled by the 35S constitutive promoter was able to restore the nodule development of *nsp2* mutants, whereas not all the nodules were completely functional. The genetic complementation of the mutant with *NSP2* confirmed that the point mutation in *NSP2* caused the nodulation deficiency of NF-FN9199; therefore, this mutant was termed *nsp2-6* hereafter. The sequence analysis of the *NSP2* gene amplified from *nsp2-6* revealed a point mutation at nucleotide position 730 in the coding sequence of *NSP2*. The single base-pair change from G to C caused an amino acid substitution of Asp to His (D244H) in VHIID, one of the five motifs of the GRAS domain of the NSP2 protein (Figure 2; Kontra-Kováts et al., 2019). The mutation in *nsp2-6* led to amino-acid change in one of the prominent residues in the so-called VHIID motif. It has been reported previously that the mutation in *nsp2-3* causes a nonconservative E232K substitution in the VHIID motif, and that a 435-bp deletion in *nsp2-2* eliminates the major part of the GRAS domain of NSP2 (Kalo et al., 2005).

### Point Mutant Alleles of *NSP2* Show Distinct Root Hair and Infection Phenotypes

**FIGURE 3:**
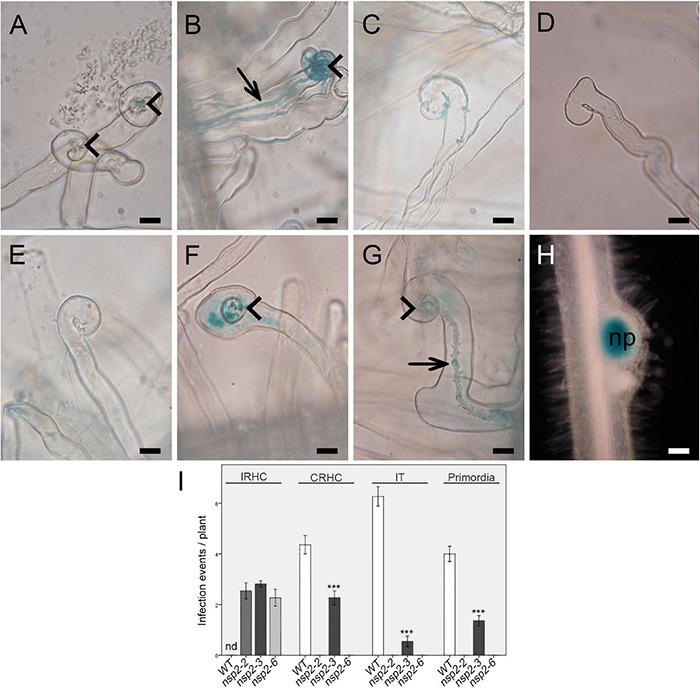
Early infection phenotype of different *nsp2* alleles. **(A)** Root hair curling at 2 days post inoculation (dpi) and **(B)** initiation of infection thread (IT) development on wild-type roots at 3 dpi with *S. medicae* strain WSM419 constitutively expressing the *gusA* gene. Rhizobia triggered merely incomplete root hair curling (IRHC) on *nsp2-2* and *nsp2-6* mutant roots at 3 dpi **(C,D)** and even at 14 dpi on *nsp2-6* roots **(E)**. Rhizobial inoculation induced complete root hair curling (CRHC) **(F)** and infection thread growth with abnormal morphology **(G)** on *nsp2-3* roots at 3 dpi and formation of nodule primordia **(H)** with infected cells at 4 dpi. Arrowhead, root hair curling; arrow, infection thread; np, nodule primordium. Scale bars: 50 μm **(A–G)** and 25 μm **(H)**. Average number of infection events detected on WT (*n* = 13) and *nsp2* mutant roots (*nsp2-2 n* = 11, *nsp2-3 n* = 15, and *nsp2-6 n* = 11) at 3 dpi and average number of nodule primordia on WT (*n* = 16) and *nsp2-3* (*n* = 16) mutant roots at 4 dpi with *S. medicae* strain WSM419 **(I)**. IRHC, incomplete root hair curling; CRHC, complete root hair curling; IT, infection thread; nd, not determined. Error bars represent standard errors. Asterisks indicate significant differences by Student’s *t*-test: ***P ≤ 0.001.

Root hair deformation and curling are the first morphological responses to rhizobial infection. The *nsp2-1* and *nsp2-2* deletion mutant alleles showed root hair deformation with imperfect curling at 3 dpi with compatible rhizobia (Oldroyd and Long, 2003). To analyze the root hair responses of the point mutant alleles of *NSP2*, *nsp2-2*, *nsp2-3*, *nsp2-6* mutant, and wild-type plants were inoculated with SmWSM419 carrying the constitutively expressed *GUS* reporter gene. We monitored the root hair responses and, in addition, the roots were stained for GUS activity to visualize rhizobia in ITs at 3 and 14 dpi. Rhizobia triggered the formation of tightly curled root hairs called shepherd’s crooks and ITs containing rhizobia on the wild-type roots (Figures 3A,B,I). The most advanced morphological alteration in the *nsp2-2* deletion mutant was the incomplete curling of root hairs without entrapped bacteria, although rhizobia were found on the surface of the root hairs (Figures 3C,I). Similar to *nsp2-2*, only imperfect curling of the root hairs could be detected in *nsp2-6* at 3 dpi (Figures 3D,I), and the monitoring of the root hairs at 14 dpi did not identify any progression in root hair responses (Figure 3E). Entrapped bacteria formed by the complete curling of root hairs on *nsp2-3* mutant roots that led to the initiation of IT growth were found in the infection chamber (Figures 3F,I). Although these ITs were often discontinuous or showed abnormal development with outgrowths and the occurrence of aggregated bacteria (Figure 3G), several ITs reached the dividing cortical cells where rhizobia were released, resulting in infected nodule primordia at 4 dpi (Figures 3H,I).

### Substitution D244H in the VHIID Motif of Nodulation Signaling Pathways 2 Impairs the Nod Factor Signaling Pathway and Blocks Nodule Organogenesis

**FIGURE 4:**
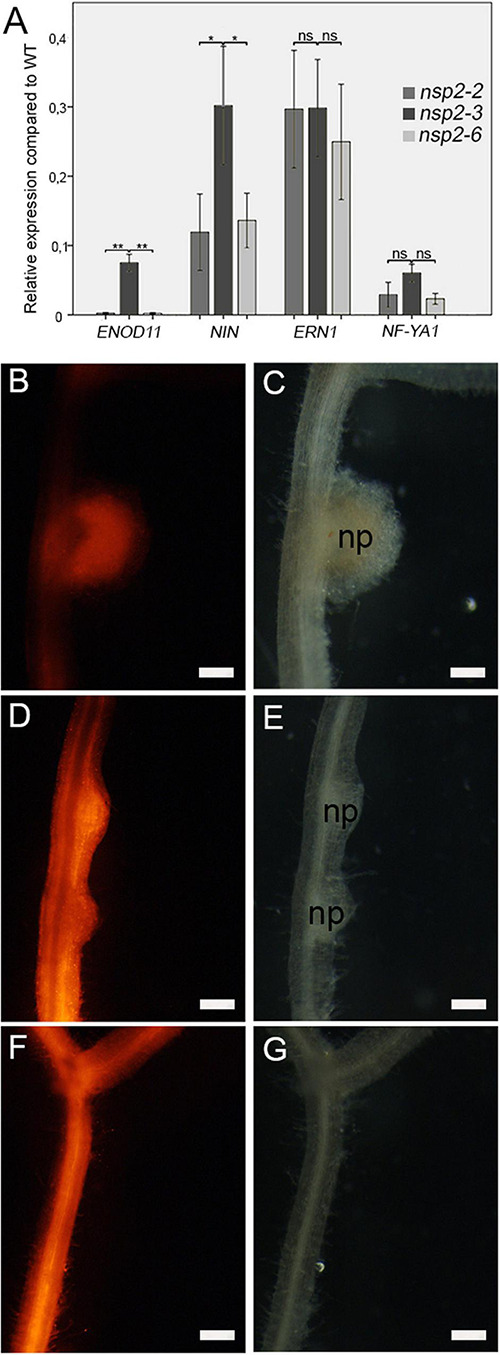
Mutant alleles of NSP2 show distinct symbiotic signaling responses. Relative transcript levels of the symbiotic marker genes *ENOD11*, *NIN*, *ERN1*, and *NF-YA1* in the *nsp2-2*, *nsp2-3*, and *nsp2-6* mutant plants were calculated in relation to those of the WT plants following inoculation with *S. medicae* strain WSM419 at 4 dpi **(A)**. Spontaneous nodule formation was induced on roots transformed with the construct of the autoactive CRE1* protein. Spontaneous nodules that developed on WT **(B,C)** transgenic roots confirmed the activity of CRE1*. The formation of nodule primordia on the *nsp2-3* **(D,E)** roots indicated that the nonconservative substitution E232K of NSP2 did not block but delayed nodule formation. Lack of spontaneous nodules on the *nsp2-6* transgenic roots **(F,G)** revealed that the substitution D244H blocked nodule organogenesis. Transgenic roots were identified by DsRed fluorescence. Values of the transcript levels are the mean of five biological replicates. Error bars represent standard errors. Asterisks indicate significant differences as determined by Student’s *t*-test: ns *P* ≥ 0.05, *P ≤ 0.05, and **P ≤ 0.01. Scale bars, 200 μm **(B–G)**.

The common symbiotic signaling pathway downstream of the CCaMK protein splits into root nodule and mycorrhiza-specific branching. NSP2 is primarily required for transmitting NF signaling to induce nodulation-specific gene expression leading to nodule organogenesis and rhizobial infection (Oldroyd and Long, 2003). To assess how point mutant alleles of NSP2 modulate the induction of root nodule-specific signaling pathway, the expression of *ENOD11*, *NIN*, *ERN1*, and *NF-YA1* (also termed Heme Activator Protein 2 gene, *HAP2*) acting downstream of the CCaMK/IPD3 and the NSP1/NSP2 regulatory complexes was monitored 4 dpi with SmWSM419 by qPCR (Oldroyd and Long, 2003; Kalo et al., 2005; Horvath et al., 2011). A severe reduction in the relative transcript level of *ENOD11*, *NIN*, and *NF-YA1* was detected in *nsp2* mutants, but the expression of these marker genes was always higher in *nsp2-3* compared with *nsp2-2* and *nsp2-6*, indicating a partial activity of NSP2_E232K_ in symbiosis-specific gene induction (Figure 4A). Although the relative expression level of *ERN1* was reduced, it did not show significant variation among the different *nsp2* alleles, suggesting a possible indirect effect of NSP2 on *ERN1* gene expression.

The formation of symbiotic nodules is the result of two coordinated developmental programs, rhizobial infection and nodule formation, and the arrest in either process blocks the progress of the other one. We demonstrated that NSP2_D244H_ blocks the infection in root hairs, but it was still in question whether the non-nodulation phenotype of *nsp2-6* was the result of the arrest of the rhizobial infection or whether the D244H substitution abolished nodule organogenesis, too. *CCaMK*, encoding a calcium- and calmodulin-dependent kinase, and the cytokinin receptor gene *CRE1* are both essential for nodule organogenesis. The activation of CCaMK and CRE1 initiates root cortical cell division and downstream signaling components, such as NSP2, which is required for the formation of nodule primordia (Gleason et al., 2006; Plet et al., 2011). The gain-of-function variants of CCAMK and CRE1 are able to induce spontaneous nodule formation (Gleason et al., 2006; Gonzalez-Rizzo et al., 2006; Tirichine et al., 2007). To test the requirements of the Asp residue at position 244 in NSP2 for nodule organogenesis, we introduced the gain-of-function version of CRE1 [CRE1^∗^ (L267F); Ovchinnikova et al., 2011]; into the roots of the wild-type (Figures 4B,C) and *nsp2* mutant plants (Figures 4D–G) by *A. rhizogenes*-mediated hairy root transformation. Transformed plants were grown in the absence of rhizobia and scored for spontaneous nodule formation 8 weeks post transformation. Primordia of spontaneous nodules formed on *nsp2-3* transgenic roots (Figures 4D,E), indicating that the mutation in *nsp2-3* is neutral for nodule induction although the NSP2_E232K_ protein variant appears to cause delayed formation of spontaneous nodules (Figures 4D,E). In contrast, no nodules could be detected on *nsp2-6* transgenic roots (Figures 4F,G), indicating that the variant NSP2_D244H_ abolishes nodule formation.

### Substitution of D244H in the VHIID Motif of Nodulation Signaling Pathways 2 Abolishes the Interaction With Nodulation Signaling Pathways 1

It was previously demonstrated in yeast and *in planta* by two-hybrid experiments and BiFC assay that NSP1 and NSP2 form heterodimers, and that this complex interact with the promoter of NF inducible genes *ENOD11*, *ERN1*, and *NIN* (Hirsch et al., 2009). It was found that the amino acid change NSP2_E232K_ in the VHIID domain in *nsp2-3* abolished the autoactivation capacity of NSP2 but did not inhibit the interaction with NSP1(Hirsch et al., 2009). To test how the substitution in mutant *nsp2-6* affects the interaction with NSP1, we tested the capability of NSP2_D244H_ to form a complex with NSP1 along with wild-type NSP2 and mutant NSP2_E232K_ in *S. cerevisiae* using a yeast-two hybrid (Y2H) system.

**FIGURE 5:**
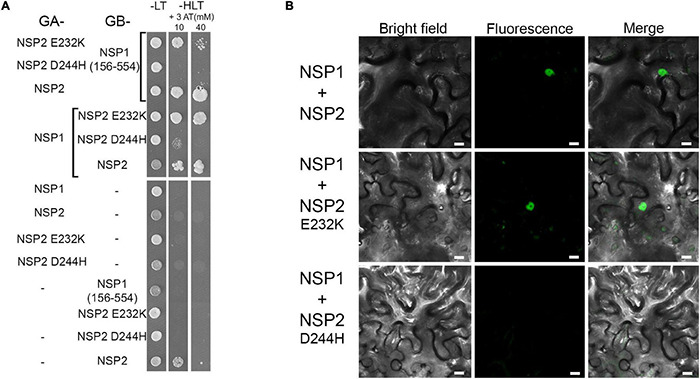
Interaction between the NSP1 and NSP2 derivatives. Interactions were tested in yeast-two-hybrid (Y2H) **(A)** and in bimolecular fluorescence complementation (BiFC) **(B)** assays. **(A)** NSP1 and NSP2 derivatives were fused either to the yeast Gal4 activation (GA) domain or the Gal4 DNA binding (GB) domain and transformed into *Saccharomyces cerevisiae* strain PJ69-4A. To test the direct interactions between NSP1 and NSP2, the growth of yeast colonies was compared in the presence (-LT) and absence [-HLT supplemented with 3-amino-1,2,4-triazole (3-AT)] of histidine. Y2H assay showed that NSP2-D244H does not interact with NSP1. In order to test the interaction between NSP1 and WT or mutant versions of NSP2 that were fused to the yeast Gal4 activation, the non-autoactive deletion version of NSP1 (156-554) fused to GB was used. **(B)** Constructs encoding for the N-terminal or C-terminal parts of the YFP fused to the N-terminal part of the NSP1 and NSP2 derivatives, respectively, were co-transformed transiently into *Nicotiana benthamiana* leaves. Interactions of the proteins in leaf epidermal cells were observed by confocal microscopy 3 days after transformation. Scale bars, 10 μm.

Nodulation Signaling Pathway 2 showed strong autoactivation in the Y2H experiments when fused to the Gal4 DNA binding domain (Hirsch et al., 2009). To improve the stringency of the interaction, we raised the amount of supplemented 3-aminotriazole (3-AT) to a relatively high concentration that abolished the autoactivation activity of NSP2 (Figure 5A), allowing us to use it in the Y2H assay. Yeast strains containing NSP2_E232K_ or NSP2_D244H_ fused to the Gal4 DNA binding domain could grow in -HLT plates, indicating the reduced capability of these proteins to autoactivate the Gal4 system (data not shown). Both wild-type NSP2 and NSP2_E232K_ interacted with NSP1, when fused either to the Gal4 DNA binding domain or to the activation domain (Figure 5A). These results are consistent with previous studies showing that NSP2_E232K_ does not affect the complex formation between NSP1 and NSP2 (Hirsch et al., 2009). However, the mutation in NSP2_D244H_ inhibited the interaction with NSP1 in yeast, indicating that the Asp residue in the VHIID motif of NSP2 was essential for the interaction with NSP1. The interaction between NSP2_E232K_ or NSP2_D244H_ with NSP1 was quantified by measuring β-galactosidase activity. The results showed a significant reduction in yeast containing NSP2_E232K_ and NSP1 (Supplementary Figure 1A) compared with the interaction between wild-type NSP2 and NSP1, although the yeast strain producing NSP2_E232K_ and NSP1 proteins could grow in the spot assay (Figure 5A). We detected low levels of β-galactosidase activity in *S. cerevisiae* producing NSP2_D244H_ and NSP1, but this activity probably did not exceed the threshold to allow the growth of yeast expressing these proteins. Control immunoblotting with anti-HA was performed to show the stability of the mutant versions of NSP2 (Supplementary Figure 2B), and that the absence of interaction between NSP2_D244H_ and NSP1 was not due to lack of the mutant NSP2 protein.

To validate the differential interaction of NSP2_E232K_ and NSP2_D244H_ with NSP1 observed in the Y2H experiment, we analyzed their association in *N. benthamiana* leaves by BiFC assay. We fused NSP1 to the N-terminal part of the yellow fluorescent protein (N-YFP) and NSP2, and NSP2_E232K_ and NSP2_D244H_ to the C-terminal part of the yellow fluorescent protein (C-YFP). We co-infiltrated the constructs of NSP1 with the different version of NSP2 into *N. benthamiana* leaf epidermal cells using *A. tumefaciens*. Previously, nuclear localization of NSP1 and NSP2 has been observed (Kalo et al., 2005; Smit et al., 2005). The nuclear signal of YFP was detected when we transiently expressed the nuclear localized NSP1 with either NSP2 or NSP2_E232K_, indicating an *in planta* interaction between these proteins (Figure 5B). No nuclear signal of YFP was detected when NSP1 was co-infiltrated with NSP2_D244H_, confirming the eliminated interaction between these proteins that has been found in Y2H previously. These results highlight the requirement of a conserved residue in the VHIID motif of NSP2 that is essential for the interaction between NSP1 and NSP2 and required for both nodule formation and rhizobial infection.

### *Medicago truncatula nsp2-3* Shows a Strain-Dependent Nodulation Phenotype

**FIGURE 6:**
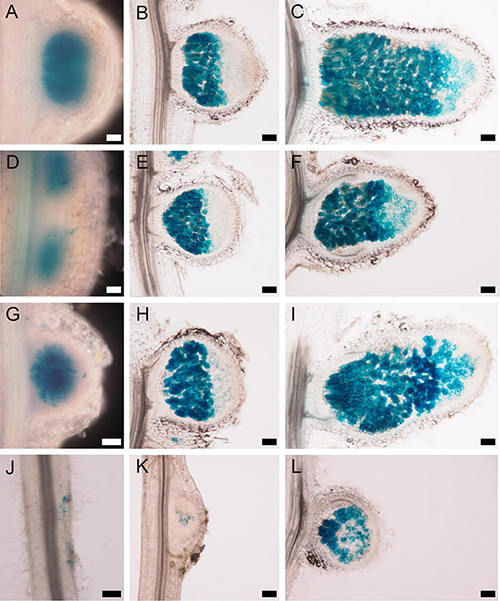
Strain-dependent nodulation kinetics of the *nsp2-3* mutant. WT *Medicago truncatula* A17 plants inoculated with either the **(A–C)** *S. medicae* strain WSM419 or with the **(G–I)** *S. meliloti* strain 1021 expressing the *lacZ* gene show similar nodulation phenotypes at **(A,G)** 4 dpi, **(B,H)** 10 dpi, and **(C,I)** 18 dpi. *nsp2-3* mutant inoculated with the *S. medicae* strain WSM419 at **(D)** 4 and **(E)** 10 dpi displayed similar nodule morphology and rhizobial colonization as WT nodules. Slightly delayed development could be detected in the *nsp2-3* nodules at **(F)** 18 dpi. *nsp2-3* mutant showed remarkable delayed nodule development at **(J)** 4 dpi, **(K)** 10 dpi, and **(L)** 18 dpi with strain *S. meliloti* 1021. Scale bars, 100 μm.

**FIGURE 7:**
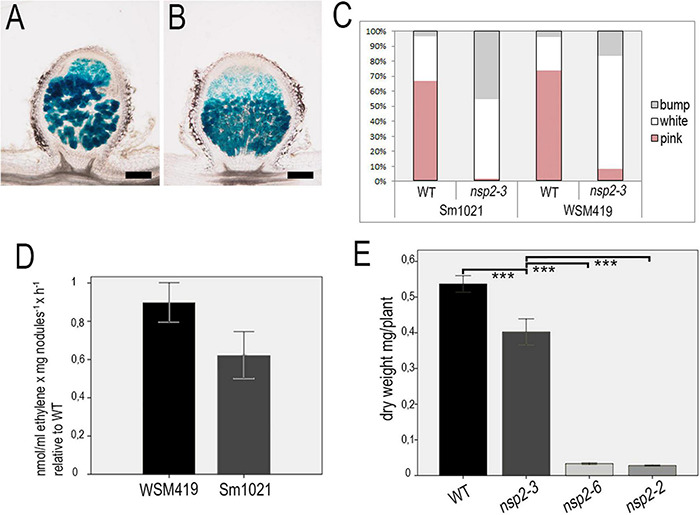
Pink nodules of the *nsp2-3* mutant plants display better symbiotic performance with SmWSM419. Colonization of the pink nodules occasionally formed on *nsp2-3* roots at 18 dpi with strains **(A)** Sm1021 or **(B)** SmWSM419 is similar. Distribution of pink (fixing) nodules, white spherical (non-fixing) nodules, and bumps (nodule primordia) that formed on WT and *nsp2-3* roots at 18 dpi **(C)**. Relative ethylene production measured by the ARA of pink nodules harvested from the *nsp2-3* and WT roots. Values represent the mean ± SE of three replicates **(D)**. Shoot dry weight of the WT and different *nsp2* mutant plants at 8 wpi with SmWSM419. Values represent the mean ± SE of 20 plants **(E)**. Asterisks indicate significant differences determined by Student’s *t*-test: ***P ≤ .001. Scale bars, 200 μm.

The emergence of nodule primordia at 4 dpi with SmWSM419 (Figure 3H) did not correspond completely to the previously reported nodulation phenotype of *nsp2-3*. The former study found no nodule primordium development on *nsp2-3* roots at 4 dpi with *S. meliloti* strain 1021 (Sm1021) although small white nodules could be detected at later time points (Kalo et al., 2005). To characterize the strain-dependent nodulation phenotype of *nsp2-3*, the kinetics of nodule development were monitored on wild-type and *nsp2-3* plants inoculated with the rhizobial strains *S. meliloti* 1021 (pXLGD4) and *S. medicae* WSM419 (pXLGD4) constitutively expressing the *lacZ* reporter gene. Nodule primordia were formed on wild-type roots at 4 dpi with both rhizobial strains and on *nsp2-3* roots inoculated with SmWSM419 (Figures 6A,D,G), but no primordia were visible on Sm1021-inoculated mutant roots at this time point (Figure 6J). Round-shaped young nodules containing invaded cells were formed on wild-type roots inoculated with both rhizobial strains and on *nsp2-3* roots (Figures 6B,E,H) at 10 dpi, but only nodule primordia were visible at this time point on *nsp2-3* roots elicited with Sm1021 (Figure 6K). Fully developed pink nodules expressing leghemoglobin and showing characteristic zonation of indeterminate nodules were found on the wild-type roots at 18 dpi with both strains (Figures 6C,I). White or occasionally slightly pinkish nodules developed on *nsp2-3* roots inoculated with SmWSM419 at 18 dpi, which were less elongated compared with wild-type nodules (Figure 6F). However, the formation of spherical undeveloped nodules on the *nsp2-3* roots at 18 dpi with Sm1021 indicated that the nodule development program was merely delayed and not blocked in the *nsp2-3* roots (Figure 6L). Infrequently, we observed pink nodules elicited by Sm1021 on the *nsp2-3* roots at 18 dpi. We counted the number of nodule primordia, and non-fixing white and nitrogen-fixing pink nodules on the wild-type and *nsp2-3* plants at 18 dpi with both rhizobial strains (Figure 7C). The wild-type plants developed a slightly higher number of functional pink nodules with SmWSM419 compared with Sm1021, confirming that *M. truncatula* genotype A17 has a better symbiotic performance with strain SmWSM419 compared with strain Sm1021 as reported previously (Terpolilli et al., 2008). The higher ratio of nodule primordia and a lower percentage of white nodules detected on the *nsp2-3* roots with Sm1021 compared with SmWSM419 indicated delayed nodule development with strain Sm1021. Although there was a difference in the percentage of leghemoglobin expressing pink nodules between the two bacterial strains, the morphology and colonization of these nodules looked similar (Figures 7A,B). To further investigate the strain-dependent nodule development and symbiotic effectiveness, the activity of bacterial nitrogenase of pink nodules that developed on the *nsp2-3* and wild-type roots was measured with the help of ARA at 3 wpi. The acetylene reduction rate of *nsp2-3* pink nodules was higher with SmWSM419 compared with Sm1021 (Figure 7D), suggesting better nitrogen fixation capacity of the interaction with strain SmWSM419. Shoot dry weight measurements of the *nsp2* mutant alleles and wild-type plants at 8 wpi with SmWSM419 showed that *nsp2-3* had significantly higher biomass than *nsp2-2* and *nsp2-6* alleles under symbiotic growth conditions (Figure 7E), indicating a weak effect of the nonconservative substitution E232K on the symbiotic function of NSP2.

### Substitution E232K in the VHIID Motif of Nodulation Signaling Pathways 2 Affects Nodule Invasion

**FIGURE 8:**
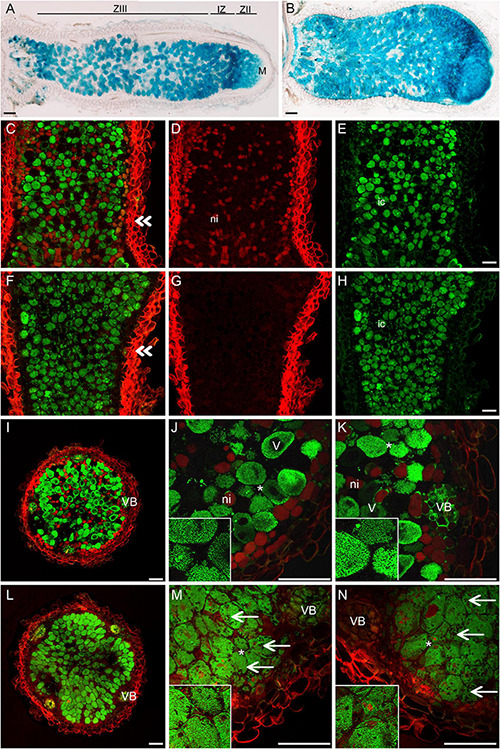
*nsp2-3* nodules contain colonized cells more densely. **(A)** WT and **(B)** *nsp2-3* plants were inoculated with SmWSM419 with the *gusA* marker gene. Pink nodules were harvested at 4 wpi, and sections were GUS-stained to detect infected cells. Sections of pink WT and *nsp2-3* mutant nodules were stained with SYTO13 and analyzed by confocal microscopy. **(C–E)** Longitudinal and cross- **(I–K)** sections from the fixation zone of WT and **(F–H)** longitudinal and cross- **(L–N)** sections from the fixation zone of *nsp2-3* nodules are shown. **(E,H)** Bacterial DNA and plant nuclei showed green fluorescence after SYTO13 staining. Outline of the nodules showed autofluorescence of the cell wall and is shown in red **(D,G)**. Panels **C** and **F** show overlay images of **(D,E)** and **(G,H)**, respectively. Panels **(J,K)** and **(M,N)** show higher magnification of cross-sections **(I,L)**. ZIII, fixation zone; IZ, interzone; ZII, invasion zone; M, meristem. Double arrowheads show the position of the cross sections, ni, non-infected cells; ic, infected cells; VB, vascular bundle; V, vacuole; arrow, starch granules in *nsp2-3* nodule cells. Scale bar, 100 μm.

The pink nodules of the *nsp2-3* mutant inoculated with SmWSM419 showed similar rhizobial colonization and nitrogen fixation capacity compared with the wild-type plants at 18 dpi, although the *nsp2-3* nodules were less elongated. The analysis of nodule morphology and rhizobial invasion at 4 wpi with SmWSM419 carrying the *GUS* reporter gene revealed that the *nsp2-3* pink nodules were thicker, and that their colonization was more extensive compared with the wild-type ones (Figures 8A,B). The structure and colonization of the *nsp2-3* pink nodules were further analyzed following staining with the nucleic acid-binding dye SYTO13 and investigation by confocal laser scanning microscopy. The autofluorescent property of cell walls was used to visualize the outline of the nodules. The analysis of the SYTO13-stained longitudinal (Figures 8C–H) and cross (Figures 8I,L) sections of 28 dpi wild-type and *nsp2-3* mutant nodules showed cells that were similarly colonized by bacteria, although some remarkable differences were observed. Similar to the GUS staining, a higher number of green fluorescent infected cells were found in the *nsp2-3* nodules (Figures 8F,L) compared with wild-type plants (Figures 8C,I), and uninfected cells displaying autofluorescence could hardly ever be observed in the *nsp2-3* nodules (Figures 8G,H,L). Higher magnification of nodule cross-sections prepared from the distal part of the nitrogen fixation zone showed infected cells containing elongated bacteroids that were orientated toward the central vacuole in the wild-type nodules (see inset images on Figure 8J,K,M,N). In the *nsp2-3* nodules, densely located infected cells with hardly visible or contracted vacuoles, disorganized symbiosomes, and tens of starch granules were detected (Figures 8M,N). The lack of autofluorescent cells in the inner cortical cell layer and uninfected cells in the central nodule tissue was also apparent in these mutant sections. The *nsp2-3* mutant nodules were extensively filled up with colonized cells but also possessed a reduced number of small uninfected cells, indicating a function of NSP2 in controlling the balance between the infected and uninfected cells. Nodule sections analyzed at higher magnification showed mostly fluorescence signals of rhizobia when analyzed in the green channel. In order to quantify the infected cells in the wild-type and *nsp2-3* mutant nodules, we measured the area of colonized cells on SYTO13-stained nodules of the *nsp2-3* and wild-type plants. Sections were analyzed using the image-analysis software FIJI (Schindelin et al., 2012) to determine the number and area of connected fluorescent regions. The analysis revealed a higher number of smaller areas of fluorescence in sections of the wild-type plants as compared with the mutant (Supplementary Figure 2) plants, indicating that the infected cells in the wild-type sections are more frequently surrounded by uninfected cells and that the mutant nodules contained more densely infected cells.

## Discussion

Two GRAS-type transcription regulators, NSP1 and NSP2, form a complex that binds to the promoters of early nodulin genes and induce their transcriptional activity (Hirsch et al., 2009). The appropriate activity of the NSP1–NSP2 complex is required for synchronized processes of IT formation and invasion of roots by nitrogen-fixing rhizobia and the induction of cortical cell divisions essential for nodule organogenesis (Catoira et al., 2000; Oldroyd and Long, 2003; Kalo et al., 2005; Smit et al., 2005).

Here, we report that two-point mutant alleles of *NSP2* containing mutation in the VHIID conserved region of the GRAS domain show distinct early responses to rhizobial inoculation. The *nsp2-6* mutant allele was identified in a screen of FN-mutagenized *M. truncatula* population (Kontra-Kováts et al., 2019). Fast neutron bombardment (FNB) is a promising source to induce smaller and larger deletions for gene disruptions. Although single base pair substitutions were detected in FN-mutagenized *Arabidopsis thaliana* populations (Belfield et al., 2012), to our knowledge, no FNB-induced single nucleotide exchange has been reported in FN-mutagenized *M. truncatula*. However, we cannot exclude that spontaneous mutation generated the nucleotide exchange in this *nsp2* allele. The mutation in *nsp2-6* caused the change in one of the conservative residues of the VHIID motif of NSP2 (NSP2_D244H_), and this mutation phenocopies the *nsp2-2* null allele identified earlier (Oldroyd and Long, 2003). Both the *nsp2-2* and *nsp2-6* alleles showed root hair deformation, but entrapped bacteria forming microcolonies were not observed when inoculated with rhizobia. These mutant alleles were similarly blocked for NF-induced gene expression. The substitution of NSP2_D244H_ abolished the interaction with NSP1 in yeast and *N. benthamiana* like the deletion derivatives of the GRAS domain of NSP2 detected previously (Hirsch et al., 2009). In addition to the deficiency in the infection process, the nodule development program was also blocked in the *nsp2-6* mutant. These phenotypes suggest that *nsp2-6* is a strong point mutant allele of *NSP2*. The VHIID motif is presumed to mediate DNA-protein interactions (Pysh et al., 1999) and maintain the structural integrity and conformation of the GRAS domain (Li et al., 2016). The symbiotic phenotype of *nsp2-6* and the abolished interaction between NSP1 and NSP2_D244H_ demonstrated the requirement of D244 in the domain of VHIID for the function of the NSP2 protein.

We also report in our study that the mutants NSP2_E232K_ and NSP2_D244H_ interact diversely with NSP1. This finding provides an explanation for the observed changes in NF-induced gene expression and distinct nodulation phenotype of *nsp2-3* and *nsp2-6*. The two point mutations occurred in the VHIID motif suggest different relevance of the residues of E232 and D244 for the function of NSP2. NSP2 and other family members of GRAS proteins contain a conserved GRAS domain that contains five motifs (LHR1, VHIID, LHRII, PFYRE, and SAW) named after their conserved residues. The analysis of the crystal structure of the GRAS domains of SCR and a SCR-LIKE protein (SCL7) (Li et al., 2016; Hakoshima, 2018) revealed that each GRAS domain comprises an a-helical cap and an a/b core subdomain; the latter contains 10 a-helices and nine b-strands (b1–b9). *nsp2-6* contains a D244H mutation in the VHIID motif, which, alone, forms the b1 strand that appears to be important for stabilizing the overall structure of the GRAS domain (Li et al., 2016; Hakoshima, 2018). The E232K mutation in *nsp2-3* is located in the a-helix A5, one of the six a-helices flanking the central b-sheet. Based on the distinct interaction of NSP2_E232K_ and NSP2_D244H_ with NSP1, we suggest that the D244H mutation affects the structure of NSP2 more seriously as compared with the E232K mutation.

In contrast to *nsp2-6*, the *nsp2-3* allele encoding NSP2 with a E232K substitution was reported to form small white nodules, although the infection and nodulation phenotype of this mutant allele has not been studied in detail (Kalo et al., 2005). Complete root hair curling and entrapment of bacteria were detected on the *nsp2-3* roots inoculated with *S. medicae* WSM419. ITs in the root hairs showed thick abnormal morphology with edgy protrusions resembling the ITs found in the root hairs of a *vapyrin* (*vpy*) mutant (Murray et al., 2011) but dissimilar to the swollen, blister-like and bulbous formations observed in *nf-ya-1* mutant root hairs (Laporte et al., 2014) or within the elongated-type nodules of *ipd3-1* (Horvath et al., 2011). However, in contrast to the mutants *vpy* and *nf-ya-1*, ITs in *nsp2-3* penetrated into the cortical cells and released bacteria, which colonized nodule primordia. Because we noticed that the development of nodule primordia was more progressed on the *nsp2-3* roots inoculated with SmWSM419 compared with the nodulation phenotype inoculated with Sm1021 (Kalo et al., 2005), the kinetics of nodule development was analyzed using a less effective (Sm1021) and a highly compatible (SmWSM419) strain (Terpolilli et al., 2008). The difference between wild-type and *nsp2-3* nodule development was not evident until 10 dpi with SmWSM419, and the reduced number of functional pink nodules at a later time point indicated the delay in nodule development. In order to analyze the performance of the pink nodules on *nsp2-3* roots elicited by SmWSM419, nitrogen fixation capacity was assayed, and dry weight measurements were conducted. The symbiotic performance of the pink nodules on the *nsp2-3* roots with SmWSM419 was nearly identical to that of the nodules of the wild-type plants, providing evidence that NSP2_E232K_ was still functioning. However, the tardy nodule differentiation, lower ratio of uninfected cells, and accumulation of starch granules in the *nsp2-3* pink nodules suggest an inaccurate action of NSP2_E232K_.

The delayed nodule development was more striking with the strain Sm1021, indicating a strain-dependent symbiotic phenotype of *nsp2-3*. The suboptimal compatibility of Sm1021 with wild-type *M. truncatula* A17 has been reported previously (Terpolilli et al., 2008; Kazmierczak et al., 2017), and the better-performing microsymbiont SmWSM419 was suggested to be used in symbiotic tests. It was found that Sm1021 efficiently induces nodule organogenesis, but that the complete differentiation of symbiotic nodule cells does not take place (Kazmierczak et al., 2017). Although the genetic basis of the reduced effectiveness of *M. truncatula* A17 with Sm1021 is still unclear, our results suggest that the strain-dependent symbiotic performance is probably already manifested in the NF signaling pathway and that an incomplete differentiation of symbiotic nodule cells or a different nitrogen-fixation capacity of these strains cannot provide explanations per se. Our results also imply the function of NF signaling components during the differentiation of nodule cells at late stages of nodulation.

The analysis of the RNA sequencing data obtained from laser-capture microdissected nodule zones (Roux et al., 2014) revealed that both *NSP1* and *NSP2* are active in the apex of mature nodule. About 70 and 90% of the *NSP1* and *NSP2* transcripts, respectively, were located in the nodule meristem and in the distal part of the infection zone (zone II), supporting a function of *NSP2* in nodule differentiation. The involvement of components of the NF signaling pathway in the late stages of the nitrogen-fixing interaction was reported earlier for *DMI2* and *NIN* (Limpens et al., 2005; Liu et al., 2021). The partial activity of both genes causes defects in symbiosome formation. Our results provide evidence that NSP2 also has a function during IT development and in mature nodules.

## Conclusion

In summary, we showed here the requirement of a conserved residue in the GRAS domain of NSP2 and the function of NSP2 during early and late stages of nodule symbiosis. Point mutations often induce a partial suppression of gene function and enable the mapping of essential residues for the activity of proteins. Our results also elucidated the benefit of studying weak alleles of symbiotic genes that can be used to define gene functions at later stages of nodule symbiosis. This study also underscores the importance of studying strain-dependent effects of early symbiotic genes.

## Data Availability Statement

The original contributions presented in the study are included in the article/Supplementary Material, further inquiries can be directed to the corresponding author.

## Author Contributions

SK, AD, and PK designed the research, and analyzed and interpreted the data. SK, LF, and AD performed the experiments. FA contributed to microscopy analysis. KL and GR conducted the acetylene reduction measurements. PK wrote the manuscript. All authors contributed to the article and approved the submitted version.

## Conflict of Interest

The authors declare that the research was conducted in the absence of any commercial or financial relationships that could be construed as a potential conflict of interest.

## Publisher’s Note

All claims expressed in this article are solely those of the authors and do not necessarily represent those of their affiliated organizations, or those of the publisher, the editors and the reviewers. Any product that may be evaluated in this article, or claim that may be made by its manufacturer, is not guaranteed or endorsed by the publisher.
