# Supplementary material for: Amino Acid Polymorphisms in the VHIID Conserved Motif of Nodulation Signaling Pathways 2 Distinctly Modulate Symbiotic Signaling and Nodule Morphogenesis in Medicago truncatula
Source: Front Plant Sci. 2021 Dec 13;12:709857. doi: 10.3389/fpls.2021.709857 (PMC8711286; doi:10.3389/fpls.2021.709857)
Supplement: Supplementary file 1 [file Data_Sheet_1.DOCX]

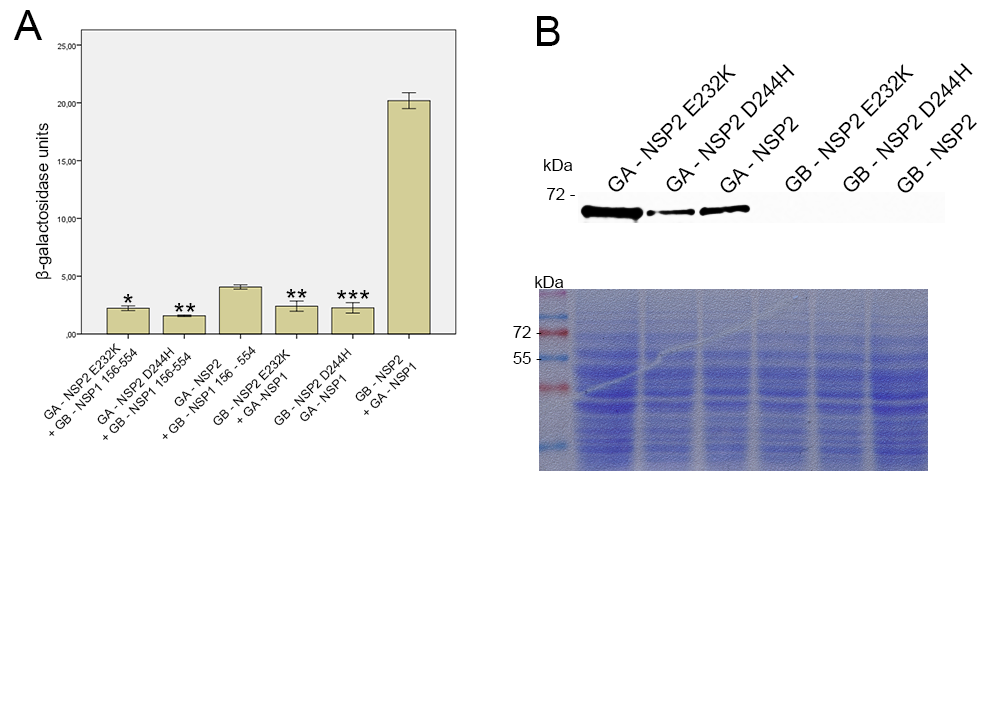


**Figure S1.** The activity of wild-type and mutant versions of NSP2 proteins quantified with measuring the β-galactosidase activity. The assay revealed an impaired interaction between NSP1 and the NSP2_E232K_/NSP2_D244H_ in *S. cerevisiae* (**A**). Wild-type and mutant forms of NSP2 proteins were fused with the Gal4 activation (GA) and DNA binding domains (GB), respectively and tested for interaction with NSP1. The non-autoactive form of NSP1 was used when it was fused to GB. The interactions were quantified using the β-galactosidase assay. Each value is the mean of three technical replicates. Error bars indicate the SE. Asterisks indicate significant differences that were determined using Student’s t test: *P ≤ 0.05, **P ≤ 0.01 and ***P ≤ 0.001.

B, Detection of wild type and mutant NSP2 proteins in yeast two-hybrid experiment using immunoblot analysis NSP2 proteins fused to the Gal4 activation domain were detected with α-HA antibody. Wild-type and mutant NSP2 proteins fused to the Gal4 DNA binding domains were used as negative controls. The same amount of proteins were loaded and stained with Coomassie blue as loading control.


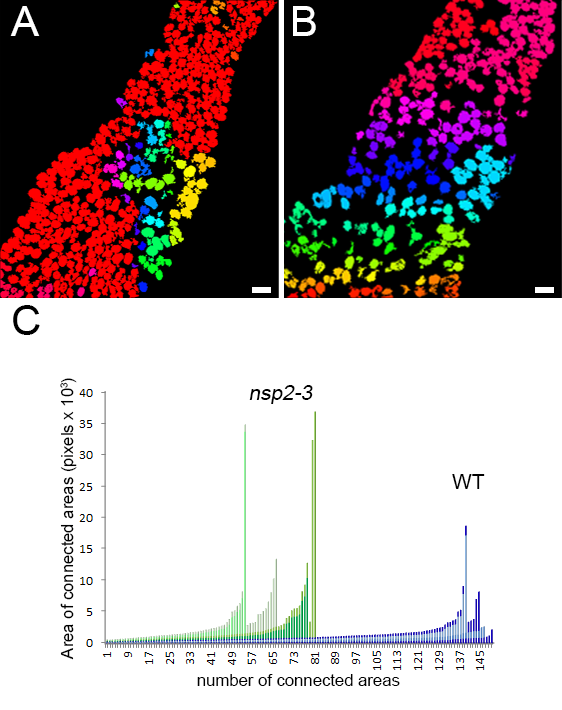


**Figure S2** The quantitative comparison of bacterial colonization in *nsp2-3* (**A**) and wild-type (**B**) nodule sections. Images of nodule sections taken with confocal laser scanning microscopy were transformed into images to highlight highly fluorescent infected-cell containing regions using the FIJI software Find connected regions plug-in. The total number and size of the connected areas were determined and plotted (**C**) for four wild-type (WT) and *nsp2-3* mutant nodule sections. Two representative images were shown in top panel in false arbitrary colors. The FIJI plug-in was originally designed for 3D connected regions, hence identified regions close to each other also colored similarly. Scale bars are 100 µm.


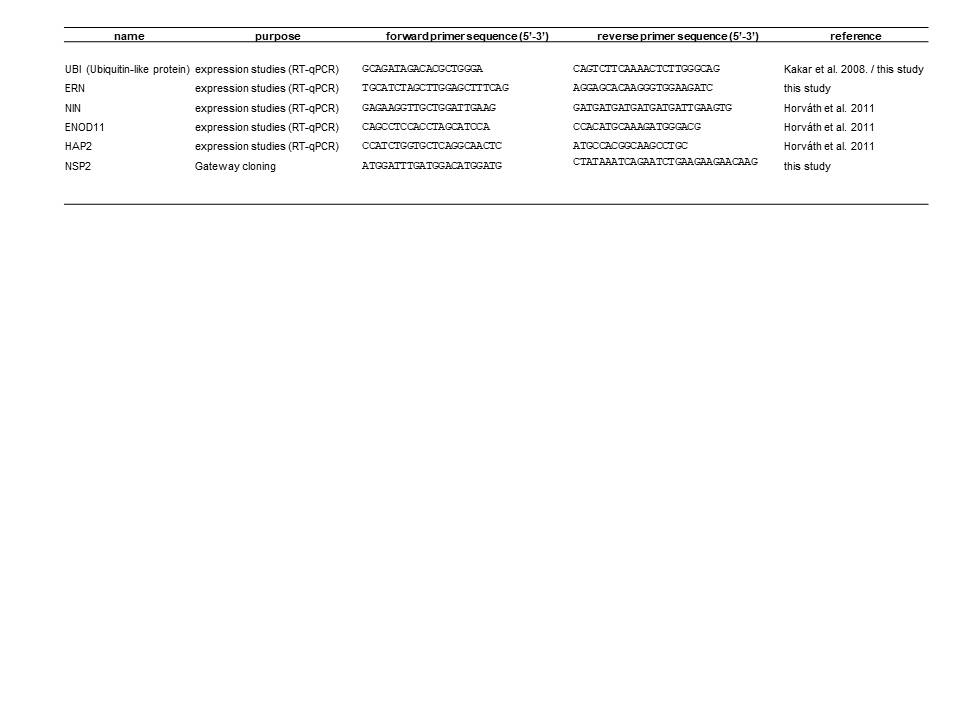


**Table S1.** Oligonucleotides used in this study
